# Are large frugivorous birds better seed dispersers than medium‐ and small‐sized ones? Effect of body mass on seed dispersal effectiveness

**DOI:** 10.1002/ece3.6285

**Published:** 2020-04-27

**Authors:** Héctor Godínez‐Alvarez, Leticia Ríos‐Casanova, Begoña Peco

**Affiliations:** ^1^ Unidad de Biología Tecnología y Prototipos Facultad de Estudios Superiores Iztacala Universidad Nacional Autónoma de México Tlalnepantla México; ^2^ Centro de Investigación en Biodiversidad y Cambio Global Departamento de Ecología Universidad Autónoma de Madrid Madrid España

**Keywords:** frequency of visits, frugivory, germination, gut retention time, quality and quantity of seed dispersal, seed removal

## Abstract

Frugivorous birds vary in seed dispersal effectiveness (SDE) depending on their body mass. It has been suggested that large birds are more effective dispersers than small ones because they consume a large number of fruits, disperse seeds of distinct sizes, and transport seeds over long distances. Yet, few studies have evaluated the impact of body mass on SDE of birds. In this study, we compiled one database for the quantity (i.e., frequency of visits to plants and number of seeds removed per visit) and quality (i.e., germination of seeds after gut passage and gut retention time of seeds) of seed dispersal by frugivorous birds to evaluate the impact of body mass on SDE. In addition, we compiled data on plant characteristics such as life‐form, fruit type, number of seeds per fruit, and size of seed to evaluate their influence on the quantity and quality of seed dispersal. Data were analyzed with linear mixed effects models and quantile regressions to evaluate the relationship between body mass of birds and quantity, quality, and SDE, in addition to the influence of plant characteristics on SDE. The body mass of birds was negatively related to the frequency of visits to plants. Furthermore, it was positively related to the number of seeds removed per visit, although negatively related to seed size. The life‐form of plants was the only factor explaining the germination of seeds after gut passage. Yet, the body mass of birds was positively related to the gut retention time of seeds. Small and medium birds have a relatively higher SDE than large birds. These results differ from the assertion that large birds are more effective dispersers of plants. Small and medium birds are also effective dispersers of plants that should be preserved and protected from the impact of human activities.

## Graphical abstract

This manuscript evaluates the effect of body mass on the seed dispersal effectiveness of birds.

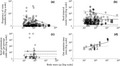

## 1 INTRODUCTION

Frugivorous birds differentially contribute to the seed dispersal of plants. Their unequal contribution to seed dispersal may be related to their abundance, digestive physiology, and body mass, among other factors (Schupp, Jordano, & Gómez, 2010). As for the body mass, small (<100 g; Jordano, García, Godoy, & García‐Cataño, 2007) and medium birds (100–500 g; Jordano et al., 2007) frequently visit plants and remove few seeds per visit. They transport seeds across short distances, decreasing the high density‐dependent mortality of seeds near mother plants and thereby contributing to the local dynamics of plant populations. Large birds (>500 g; Jordano et al., 2007) make fewer visits to plants but remove many seeds per visit. They transport seeds long distances, facilitating the colonization of new sites and thereby contributing to the metapopulation dynamics of plants (Jordano et al., 2007; Li, Li, An, & Lu, 2016; Schupp et al., 2010; Spiegel & Nathan, 2007).

The body size of frugivorous birds therefore influences the quantity and quality of seed dispersal and, accordingly, the seed dispersal effectiveness (SDE, sensu Schupp et al., 2010). However, few studies have evaluated the effect of body mass on SDE of birds (Dennis & Westcott, 2007; Jordano et al., 2007; Larsen & Burns, 2012; Li et al., 2016; Mokotjomela, Downs, Esler, & Knight, 2016; Spiegel & Nathan, 2007). One study found that the SDE of medium birds was similar to that of small birds, although differences existed in the quantity and quality of seed dispersal (Spiegel & Nathan, 2007). For example, small birds had a higher frequency of visits than medium birds. However, medium birds had longer duration of visits and gut retention time as well as larger flight distance than small birds (Spiegel & Nathan, 2007). Other studies have suggested that SDE would be positively related to body mass because large birds remove larger number of seeds and have longer gut retention times than small birds (Jordano, 2000; Mokotjomela et al., 2016; Nathan et al., 2008; Schurr et al., 2009).

To better understand the effect of body mass on SDE, it is necessary to evaluate differences in the quantity and quality of seed dispersal among frugivorous birds of different size or weight. This evaluation can help to determine whether large birds are more effective seed dispersers than medium or small birds (Jordano et al., 2007; Mokotjomela et al., 2016; Spiegel & Nathan, 2007). Some studies have suggested that large birds are effective dispersers because they consume a large number of fruits (Jordano, 2000; Jordano & Schupp, 2000; Spiegel & Nathan, 2007), disperse seeds of distinct sizes (Chen & Moles, 2015), and transport seeds long distances (Nathan et al., 2008; Schurr et al., 2009; Wotton & Kelly, 2012). However, these studies have separately evaluated data on the quantity and quality of seed dispersal. It is necessary to carry out a more complete evaluation that jointly considers both the quantity and quality of seed dispersal.

In the present study, we aimed to evaluate the effect of body mass on the SDE of frugivorous birds. We decided to only evaluate frugivorous birds because they are a well‐known group, and a good amount of data exists on the quantity and quality of seed dispersal (see Data accessibility section), body mass (del Hoyo et al., 2019; Encyclopedia of Life, 2019), and food habits (del Hoyo et al., 2019; Encyclopedia of Life, 2019) of this group compared to other frugivores such as reptiles and mammals. The focus on frugivorous birds also ensures that data on the quantity, quality, and effectiveness of seed dispersal are relatively homogeneous and comparable among species.

**FIGURE 1:**
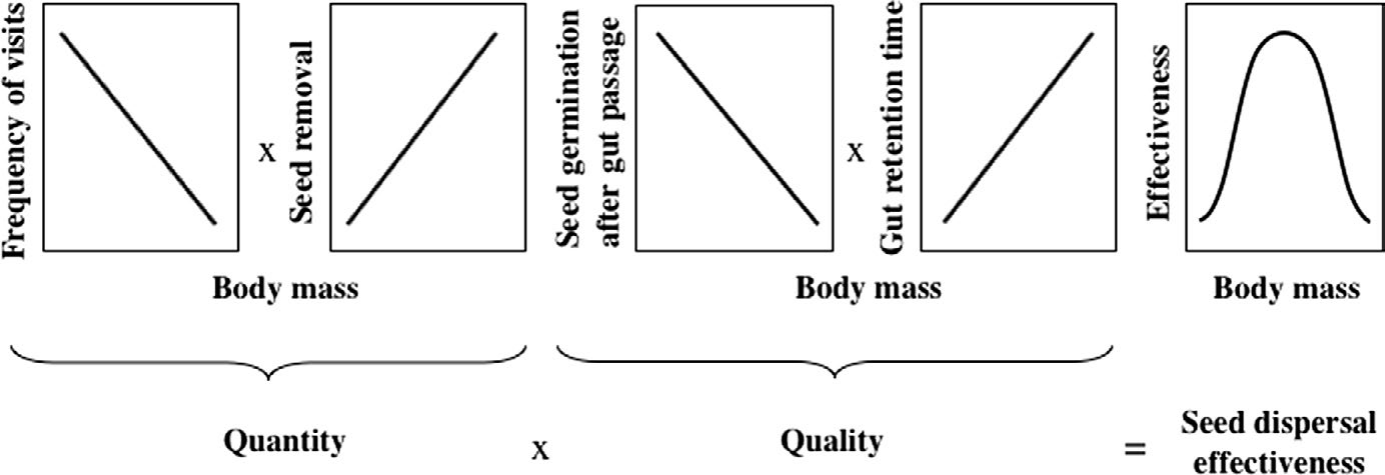
Conceptual diagram showing the estimation of seed dispersal effectiveness along with the hypothetical relationships between the body mass of birds and the quantity (i.e., frequency of visits and seed removal), quality (i.e., germination after gut passage and gut retention time), and effectiveness (i.e., quantity multiplied by quality)

To accomplish our goal, we assumed that the quantity (i.e., frequency of visits to plants and number of seeds removed per visit) and quality (i.e., germination of seeds after gut passage and gut retention time of seeds) of seed dispersal are differentially related to body mass (Figure 1), as previously reported in the ecological literature. Specifically, we assumed that the frequency of visits to plants would be negatively related to body mass (Li et al., 2016; Spiegel & Nathan, 2007) and that the number of seeds removed per visit would be positively related to body mass (Jordano, 2000; Jordano & Schupp, 2000; Spiegel & Nathan, 2007). The germination of seeds after gut passage would be negatively related to body mass (Pérez‐Méndez, Rodríguez, & Nogales, 2018; Traveset & Verdú, 2002), and the gut retention time (GRT) would be positively related to body mass (Karasov, 1990; Mokotjomela et al., 2016; Nathan et al., 2008; Schurr et al., 2009; Spiegel & Nathan, 2007). Lastly, we assumed that the SDE, calculated as the product of the quantity and quality of seed dispersal, would be greater in medium birds than in small and large birds (Mokotjomela et al., 2016; Schupp, 1993), because the first consistently provide intermediate values of quantity and quality whereas the second and third only provide low or high values of quantity or quality.

To test these hypotheses, we reviewed studies on these topics published in the last 33 years to obtain data on the quantity and quality of seed dispersal by frugivorous birds. In addition, we obtained data on plant characteristics (i.e., life‐form, fruit type, number of seeds per fruit, and size of seed) that influence the quantity and quality of seed dispersal (Jordano, 2000; Traveset & Verdú, 2002; Traveset et al., 2007; Mokotjomela et al., 2016) from published studies and online databases. We evaluated the statistical relationship between the body mass of frugivorous birds and the quantity of seed dispersal, quality of seed dispersal, and SDE, in addition to the influence of plant characteristics on these relationships.

## 2 MATERIALS AND METHODS

### 2.1 Data compilation

Studies on seed dispersal were searched in the ISI Web of Knowledge from 1985 to June 2018 using the terms “seed dispers*” or “seed remov*” or “frugivor*” and “fleshy fruit*,” excluding “herbivore*” or “dry fruit*”. We did not consider herbivores or dry fruits because herbivores consume dry fruits unintentionally (Chen & Moles, 2015). Because of this unintentional fruit consumption, data on the frequency of visits to the plants and the number of seeds removed per visit are practically nonexistent. Therefore, data are incomplete and we cannot evaluate the seed dispersal effectiveness. Furthermore, the SDE framework emphasizes endozoochorus dispersal of fleshy fruited plants (Schupp et al., 2010). We found 2,670 studies with data specific to seed dispersal by insects, fish, birds, reptiles, and mammals. Of these studies, we only selected those that provided data on the quantity (frequency of visits and seed removal) and quality (seed germination after gut passage and GRT) of the seed dispersal of wild, native, or exotic plants in natural environments by frugivorous birds. We did not consider cultivated plants or transformed environments, such as crop fields or tree plantations, because we were interested in natural patterns of seed dispersal. To obtain data on seed germination after gut passage and GRT, we considered field and laboratory studies as well as feeding experiments in captivity. According to these criteria, we found 49 studies (Appendix 1) describing 233 plant–bird interactions with data on frequency of visits (Appendix 2), 240 interactions with data on seed removal (Appendix 3), 49 interactions with data on seed germination (Appendix 4), and 34 interactions with data on the GRT of seeds (Appendix 5).

To ensure that the data on the quantity and quality of seed dispersal were comparable, the frequency of visits was calculated as the number of visits per hour and seed removal as the number of seeds removed per visit. When seed removal was reported as the number of fruits removed per visit, we multiplied the number of fruits by the number of seeds per fruit to calculate the number of seeds per visit. The number of seeds per fruit for each plant species was obtained from several information sources as described below. Seed germination after gut passage was calculated as the odds ratio based on the germination probability of defecated seeds divided by the germination probability of intact seeds. In both cases, defecated seeds and intact seeds, the germination probability was calculated as the number of germinated seeds divided by the number of nongerminated seeds (Traveset & Verdú, 2002). We did not consider the germination of regurgitated seeds because these seeds did not pass through the gut of birds. Also, we did not consider the germination of seeds with pulp to avoid the potential inhibitory effect of pulp on germination. Finally, the GRT was calculated in minutes.

In addition to these data, we also determined for each plant species the life‐form (tree, shrub, or herb), type of fruit (aril, drupe, or berry), number of seeds per fruit, and seed size (length [mm], width [mm], or weight [mg]). The measurements of length, width, and weight of seeds were used to calculate the volume of seeds with the equations reported by Chen and Moles (2015). We calculated the volume of seeds because it is related to the variety of birds that are able to consume the seeds (Jordano, 2000; Muñoz, Schaefer, Böhning‐Gaese, & Schleuning, 2017), the space occupied in the gut (Jordano, 2000), and the retention time in the gut (Fukui, 2003), which influence the quantity and quality of seed dispersal. The life‐form, type of fruit, number of seeds per fruit, and seed size were obtained from the source papers, papers cited by the source papers, online flora databases, online species description, the Seed Information Database (Royal Botanic Gardens Kew, 2019), and the Encyclopedia of Life (2019). For each bird species, we determined the body mass (g) which was obtained from the source papers, papers cited by the source papers, online bird databases, online species description, the Encyclopedia of Life (2019), and the Handbook of the Birds of the World Alive (del Hoyo et al., 2019). We determined the body mass because it is correlated with morphological characteristics such as gape width (Jordano, 2000; Moran & Catterall, 2010), gut length (Caviedes‐Vidal et al., 2007), and gut capacity (Karazov, 1990; Jordano, 2000), which influence the quantity and quality of seed dispersal.

### 2.2 Data analysis

The frequency of visits, seed removal, seed germination after gut passage, and GRT were analyzed with linear mixed effects models, in which the body mass of birds, seed number, and seed size were considered as covariates, the life‐form of plants and type of fruit were considered as fixed factors, and the study from which data were obtained was considered as a random factor. We evaluated six models to describe the relationship between the explanatory variables and each of the response variables. The model 1 only included the intercept (y = a). The model 2 included the intercept and the effect of body mass (y = a + body mass). The model 3 included the intercept, the effect of body mass, the effect of life‐form, and their interaction (y = a + body mass + life‐form + body mass × life‐form). The model 4 included the intercept, the effect of body mass, the effect of type of fruit, and their interaction (y = a + body mass + type of fruit + body mass × type of fruit). The model 5 included the intercept, the effect of body mass, the effect of seed number, and their interaction (y = a + body mass + seed number + body mass × seed number). The model 6 included the intercept, the effect of body mass, the effect of seed size, and their interaction (y = a + body mass + seed size + body mass × seed size). We did not fit models with the interaction of three or more variables because for particular combinations of variables there were no enough data to confidently estimate the coefficients of interaction. The criterion used to select the best model was the Akaike information criterion (AIC). According to this criterion, the best model was the one that has the minimum AIC among all the models. When there were no significant differences in the AIC among several models, we selected the simplest model (i.e., the model with main effects was preferred over the model with additive effects, which in turn was preferred over the model with multiplicative effects) in which all coefficients were significant.

The frequency of visits and seed removal were also analyzed by quantile regressions (5th and 95th quantiles) to quantify the direction and strength of the relationships between the explanatory variables and the response variables. The standard errors of the coefficients were estimated with bootstrapping because the sample size was low. The seed germination after gut passage was not analyzed by quantile regression because the results showed that it was not significantly related to the body mass of birds. The GRT was not analyzed by quantile regression either because the sample size was low to confidently estimate the standard errors of the coefficients, even with bootstrapping.

All data were logarithmically transformed (log [x + 1]) to comply with the assumptions of normality and homoscedasticity. After fitting the linear mixed effects models, we checked the residual plots to confirm these assumptions. All statistical analyses were carried out in R version 3.5.1 (R Core Team, 2018) using the stats (R Core Team, 2018), nlme (Pinheiro et al., 2019), and quantreg (Koenker, 2018) programs.

The values predicted by the linear mixed effects models for the frequency of visits and seed removal (quantity of seed dispersal) as well as for the GRT (quality of seed dispersal) were multiplied to calculate the SDE. The seed germination after gut passage was not included in the calculation of SDE because it was not significantly related to the body mass of birds. The relationship between the body mass of birds and the SDE was fitted to a polynomial regression model with JMP version 13.2 (SAS Institute, 2016).

## 3 RESULTS

### 3.1 Frequency of visits

**FIGURE 2:**
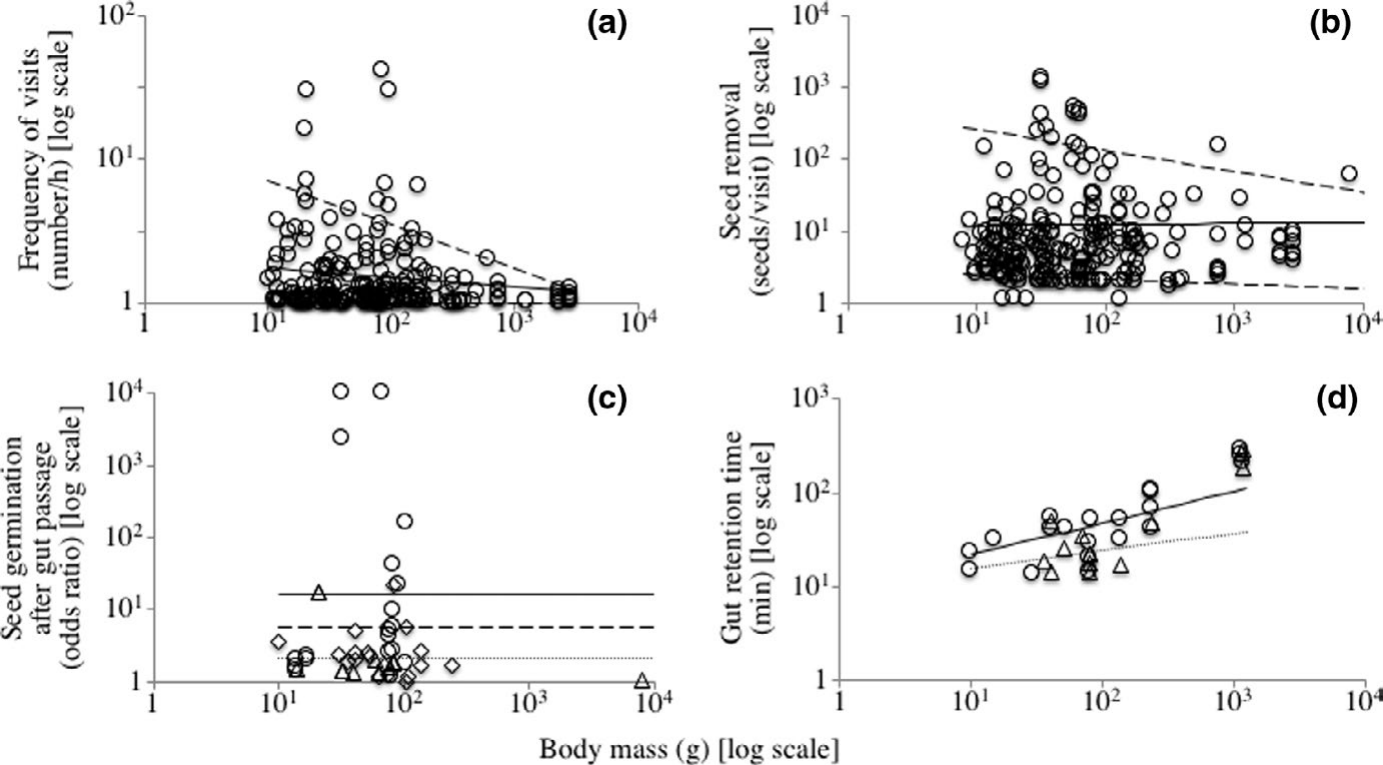
Relationship between the body mass of birds and the frequency of visits (a) seed removal (b) seed germination after gut passage (c) and gut retention time (d). For the frequency of visits and seed removal, the continuous line shows the linear regression and the discontinuous line shows the 5th and 95th quantile regressions. For the seed germination, the triangle and discontinuous line refer to herbs, the circle and continuous line refer to shrubs, and the diamonds and dotted line refer to trees. For the gut retention time, the circle and continuous line show the observed values and linear regression for berry fruit, whereas the triangle and dotted line show the observed values and linear regression for drupe fruit, respectively

The best model for the frequency of visits only included the body mass of birds (Table 1). The relationship between these variables was negative (*F*_1, 216_ = 7.275, *p* = .008; Table 2). The upper limit of the frequency of visits was also negatively related to the body mass of birds (95th percentile: slope = −0.307, *p* = .002). However, the lower limit did not differ from zero (5th percentile: slope = −0.000, *p* = .856; Figure 2a).

**TABLE 1:** Models explored in the analysis of quantity (i.e., frequency of visits and seed removal) and quality (i.e., seed germination after gut passage and gut retention time) of seed dispersal effectiveness and their Akaike's information criterion (AIC). The selected models are indicated in italics

Model	AIC
Frequency of visits	
Intercept	−96.497
*Body mass*	*−101.715*
Body mass × Life‐form	−101.695
Body mass × Fruit type	−95.758
Body mass × Seeds/fruit	−99.142
Body mass × Seed size	−97.984
Seed removal	
Intercept	265.277
Body mass	250.436
Body mass × Life‐form	249.965
Body mass × Fruit type	237.140
Body mass × Seeds/fruit	247.169
Body mass × Seed size	228.062
*Body mass + Seed size*	*228.937*
Seed germination after gut passage	
Intercept	96.550
Body mass	97.906
Body mass × Life‐form	94.830
Body mass + Life‐form	92.098
*Life‐form*	*91.014*
Body mass × Fruit type	101.449
Body mass × Seeds/fruit	101.459
Body mass × Seed size	101.802
Gut retention time	
Intercept	9.180
Body mass	2.495
Body mass × Life‐form	−1.323
Body mass × Fruit type	−0.530
*Body mass + Fruit type*	*−0.180*
Body mass × Seeds/fruit	4.335
Body mass × Seed size	3.227

**TABLE 2:** Regression coefficients of the models used in the analysis of quantity (i.e., frequency of visits and seed removal) and quality (i.e., seed germination after gut passage and gut retention time) of seed dispersal effectiveness

Model	*N*	Coefficient	*p*
Frequency of visits			
Intercept	216	0.315	.000
Body mass	216	−0.067	.008
Seed removal			
Intercept	214	0.808	.000
Body mass	214	0.311	.000
Seed size	214	−0.246	.000
Seed germination after gut passage			
Intercept_herb_	31	0.770	.017
Intercept_shrub_	31	1.211	.061
Intercept_tree_	31	0.324	.233
Gut retention time			
Intercept	22	1.014	.000
Body mass_berry_	22	0.332	.000
Body mass_drupe_	22	0.186	.043

### 3.2 Seed removal

The best model for seed removal included the body mass of birds and seed size (Table 1). The body mass of birds was positively related to seed removal (*F*_1, 214_ = 18.588, *p* < .000) whereas seed size was negatively related (*F*_1, 214_ = 26.106, *p* < .000; Table 2). Similarly, the upper limit of seed removal was positively related to the body mass of birds (95th percentile: slope = 0.572, *p* = .000) and negatively related to seed size (95th percentile: slope = −0.722, *p* = .000). The lower limit however did not differ from zero for either the body mass of birds (5th percentile: slope = −0.016, *p* = .879) or seed size (5th percentile: slope = −0.039, *p* = .526; Figure 2b).

### 3.3 Seed germination after gut passage

The body mass of birds was not related to the germination of seeds after gut passage (Table 1). The only explanatory variable included in the model was the life‐form of plants (F_2, 31_ = 4.956, *p* = .014; Table 2). The germination was higher for shrubs than for trees (*p* = .010). The germination was intermediate for herbs and did not differ from shrubs (*p* = .061) and trees (*p* = .233; Figure 2c).

### 3.4 Gut retention time

The best model for GRT included the body mass of birds and type of fruit (Table 1). The body mass of birds was positively related to the GRT (*F*_1, 22_ = 12.951, *p* = .002; Figure 2d). However, this relationship was modified by the type of fruit (*F*_1, 22_ = 4.604, *p* = .043). The slope of the regression was higher for berry fruit than for drupe fruit (Table 2). The effect for aril fruit was not evaluated because of the low amount of data.

### 3.5 Seed dispersal effectiveness

**FIGURE 3:**
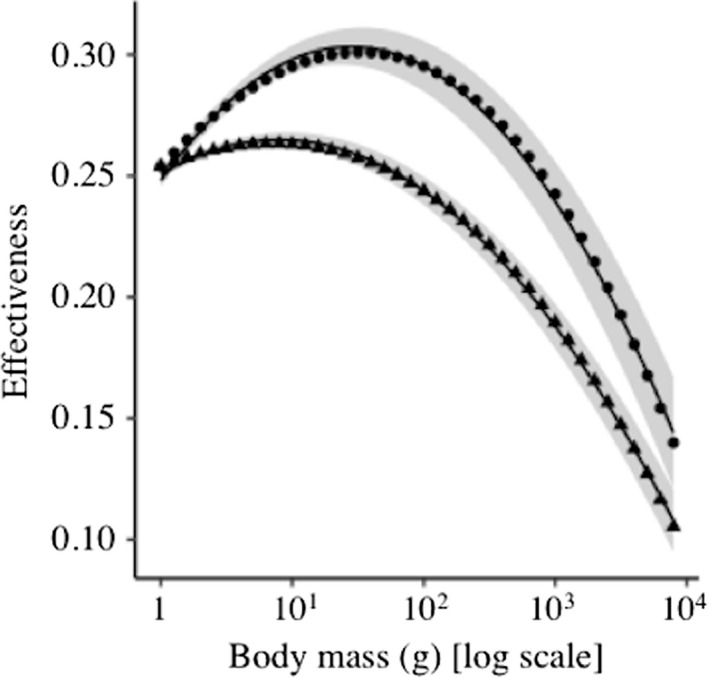
Relationship between the body mass of birds and the seed dispersal effectiveness for the berry (circles) and drupe (triangles) fruits. The continuous line shows the model fitted to data [Berry: Effectiveness = 0.248 + (0.077 × body mass) − (0.026 × body mass^2^), *R* ^2^ = 0.99; Drupe: Effectiveness = 0.251 + (0.032 × body mass) − (0.018 × body mass^2^), *R* ^2^ = 0.99] and the ribbon shows the 95% confidence intervals

The SDE increased with the body mass of birds until reaching a maximum between 10 and 100 g and then decreased until reaching a minimum at 10,000 g (Figure 3). This relationship was relatively similar for the berry (effectiveness = 0.248 + (0.077 × body mass) − (0.026 × body mass^2^), *R* ^2^ = 0.99) and drupe (effectiveness = 0.251 + (0.032 × body mass) − (0.018 × body mass^2^), *R* ^2^ = 0.99) fruits.

## 4 DISCUSSION

The aim of this study was to evaluate the effect of body mass on the SDE of frugivorous birds. It is important however to bear in mind that our searching criteria might have excluded some studies focusing on fleshy fruits that mentioned the words herbivore or dry fruit in the text, thus limiting and biasing the number of plant–bird interactions used to evaluate the relationship between the body mass of birds and the quantity, and quality of seed dispersal.

### 4.1 Frequency of visits and seed removal

In accordance with our assumptions, the body mass of birds was negatively related to the frequency of visits. Furthermore, the body mass of birds was positively related to seed removal whereas seed size was negatively related. These results suggest that large birds visit plant less frequently, but remove a higher number of seeds per visit, than medium and small birds. Seed removal however is higher for small seeds than for large seeds. Similarly, other authors have found positive relationships between the body mass of birds and seed removal (Jordano, 2000) or fruit removal (Muñoz et al., 2017). Muñoz et al. (2017) also found that fruit size is negatively related to fruit removal.

Differences in the frequency of visits and seed removal among birds of distinct body mass might be related to gape width, gut capacity, and retention time. Gape width is positively related to body mass in birds (Moran & Catterall, 2010). Thus, birds with large gape widths consume a higher number of fruits (Moran & Catterall, 2010; Naniwadekar, Chaplod, Datta, Rathore, & Sridhar, 2019) and drop a lower number of fruits (Jordano & Schupp, 2000; Naniwadekar et al., 2019) during feeding bouts than birds with small gape widths. Similarly, gut capacity and retention time are positively related to body mass in birds (Karazov, 1990; Jordano, 2000; Schurr et al., 2009; Tsoar, Shohami, & Nathan, 2011). Thus, small birds ingest few fruits and retain them for less time in the gut than large birds. This short retention time determines that small birds visit plants more frequently to feed. Yet, the number of seeds that small birds can disperse is low. Besides the influence of these morphological and physiological characteristics, the differences in the frequency of visits and seed removal among birds of distinct body mass might be related to the duration of visits (Muñoz et al., 2017) and aggressive interactions among species (Herrera & Jordano, 1981; Martin, Freshwater, & Ghalambor, 2017). The aggressive interactions occur when birds of distinct body mass compete for a limited number of fruits. Large birds displace medium and small birds through aggressive behavior. Therefore, the duration of their visits and the number of seeds removed are higher (Herrera & Jordano, 1981; Muñoz et al., 2017).

### 4.2 Seed germination after gut passage and gut retention time

Contrary to our assumptions, the body mass of birds was not related to seed germination. The life‐form of plants was the only factor influencing the germination of seeds after gut passage. The germination was lower in trees than in herbs and shrubs. These results suggest that seeds of different life‐forms of plants have characteristics that make them more or less vulnerable to gut passage. Other authors have also found that germination of trees was more affected by gut passage than other life‐forms of plants (Traveset, 1998; Traveset & Verdú, 2002). Seeds have some characteristics such as volume and strength of the coat which determine their vulnerability to gut passage in waterbirds (Kleyheeg, Claessens, & Soons, 2018). However, it is unknown whether these characteristics are related to the life‐form of plants. Similarly, seed dormancy is probably related to the life‐form of plants in temperate zones (Traveset & Verdú, 2002), but its influence on the vulnerability of seeds to gut passage has not been evaluated yet.

The body mass of birds was positively related to the GRT of seeds. These results suggest that large birds had higher GRT than medium and small birds. Other authors have also found that GRT is positively related to body mass not only in birds (Jordano, 2000; Karazov, 1990; Mokotjomela et al., 2016; Nathan et al., 2008; Schurr et al., 2009; Wotton & Kelly, 2012; Yoshikawa, Kawakami, & Masaki, 2019) but also in fishes, reptiles, and mammals (Yoshikawa et al., 2019). Differences in the GRT among birds of distinct body mass might be related to the length of gut. The length of gut is positively related to body mass in birds (Caviedes‐Vidal et al., 2007). Thus, large birds might retain fruits for longer time in the gut than small birds. Our results also showed that the GRT is higher for berry fruit than for drupe fruit. These differences are related to the seed size of these fruits. The berry fruit has a high number of small seeds, whereas the drupe fruit has a single large seed. The GRT is higher for small seeds than for large seeds (Fukui, 2003).

The higher GRT of seeds by large birds can increase the probability that seeds are dispersed long distances. Different authors have found that GRT is positively related to dispersal distance (Jordano, 2000; Nathan et al., 2008; Schurr et al., 2009; Tsoar et al., 2011). Therefore, seeds ingested by large birds are dispersed greater distances than seeds ingested by medium or small birds. The seed dispersal of plants over long distances in fragmented environments can increase the probability that plants colonize new sites (Schurr et al., 2009; Spiegel & Nathan, 2007; Tsoar et al., 2011). However, the colonization of new sites also depends on the successful establishment of plants after seed dispersal. Hyatt et al. (2003) reviewed the effect of dispersal distance on the fate of seeds and found that dispersal over long distances did not significantly increase survival; thus, it is possible in some cases that seed dispersal over longer distances impedes seedling establishment.

### 4.3 Seed dispersal effectiveness and body mass of birds

The SDE was greater in small birds than in medium and large birds. The high effectiveness of small birds was due to their high frequency of visits but low seed removal and retention time (e.g., Spiegel & Nathan, 2007). Meanwhile, the low effectiveness of large birds was due to their high seed removal and GRT but low frequency of visits (e.g., Mokotjomela et al., 2016; Schurr et al., 2009). These results support the claim that the quantity and quality of seed dispersal are inversely related, thereby bird species that ingest a high number of fruits and deposit viable seeds far from the mother plant are not necessarily the most effective seed dispersers (Schupp et al., 2010).

The relatively high SDE of small and medium birds suggests that these birds are better seed dispersers than large birds. However, under natural conditions, seeds can be ingested by different bird species with distinct SDE that complementarily disperse seeds. In the case that multispecies dispersal is advantageous for a plant species, evolution will likely favor interactions with numerous seed dispersers instead of specialization in seed dispersal or reliance on the most effective disperser (Schupp et al., 2010). Based on these assertions, small and medium birds disperse a small amount of seeds to sites within populations, thereby contributing to the local dynamics of plant populations. Meanwhile, large birds disperse many seeds to sites far from the mother plant, thereby contributing to the metapopulation dynamics of plants (Schurr et al., 2009; Spiegel & Nathan, 2007; Tsoar et al., 2011).

The relatively high effectiveness of small and medium birds in our study contrasts with the assertion that large birds are the most effective seed dispersers of plants. It was previously suggested that, because large birds are effective seed dispersers yet also highly susceptible to human activities, conservation efforts should be focused on these birds (Sekercioglu, Daily, & Ehrlich, 2004; Wenny et al., 2011). However, up until now, studies on seed dispersal by large birds have only evaluated some aspects of the quantity and quality of seed dispersal, such as seed removal, germination, and dispersal distance (Bravo, Velilla, Bautista, & Peco, 2014; Holbrook & Smith, 2000; Wotton & Kelly, 2011, 2012). Few studies have simultaneously evaluated the quantity and quality of seed dispersal (Li et al., 2016; Mokotjomela et al., 2016; Spiegel & Nathan, 2007). Our results show that small and medium birds are effective seed dispersers of plants, and therefore, it is also important to protect these birds from the impact of human activities and conserve them. In fact, the conservation of small and medium birds may imply less effort because these birds are more abundant and less sensitive to disturbance than large birds (Breitbach, Laube, Steffan‐Dewenter, & Bohning‐Gaese, 2010; Moore & Swihart, 2007).

## CONFLICT OF INTERESTS

None.

## AUTHOR CONTRIBUTION

**Héctor Godínez‐Alvarez**, **Leticia Ríos‐Casanova**, and **Begoña Peco**: Conceptualization and design of review. **Héctor Godínez‐Alvarez** and **Leticia Ríos‐Casanova**: Data acquisition; data analysis; writing—first draft of manuscript. **Begoña Peco**: Draft editing.

## Data Availability

The data supporting the analyses are available from figshare:

## Appendix 1

https://doi.org/10.6084/m9.figshare.12048885.v1

## Appendix 2

https://doi.org/10.6084/m9.figshare.12048882.v1

## Appendix 3

https://doi.org/10.6084/m9.figshare.12048894.v1

## Appendix 4

https://doi.org/10.6084/m9.figshare.12048891.v1

## Appendix 5

https://doi.org/10.6084/m9.figshare.12048888.v1
